# Opportunities for reducing emergency diagnoses of colon cancer in women and men: A data‐linkage study on pre‐diagnostic symptomatic presentations and benign diagnoses

**DOI:** 10.1111/ecc.13000

**Published:** 2019-02-08

**Authors:** Cristina Renzi, Georgios Lyratzopoulos, Willie Hamilton, Bernard Rachet

**Affiliations:** ^1^ Department of Behavioural Science and Health University College London London UK; ^2^ Cancer Survival Group, Department of Non‐communicable Disease Epidemiology London School of Hygiene and Tropical Medicine London UK; ^3^ University of Exeter Medical School Exeter UK

**Keywords:** colon cancer, data‐linkage, emergency diagnosis, primary care, symptoms

## Abstract

**Objectives:**

To identify opportunities for reducing emergency colon cancer diagnoses, we evaluated symptoms and benign diagnoses recorded before emergency presentations (EP).

**Methods:**

Cohort of 5,745 colon cancers diagnosed in England 2005–2010, with individually linked cancer registry and primary care data for the 5‐year pre‐diagnostic period.

**Results:**

Colon cancer was diagnosed following EP in 34% of women and 30% of men. Among emergency presenters, 20% of women and 15% of men (*p* = 0.002) had alarm symptoms (anaemia/rectal bleeding/change in bowel habit) 2–12 months pre‐diagnosis. Women with abdominal symptoms (change in bowel habit/constipation/diarrhoea) received a benign diagnosis (irritable bowel syndrome (IBS)/diverticular disease) more frequently than men in the year before EP: 12% vs. 6% among women and men (*p* = 0.002). EP was more likely in women (OR = 1.20; 95% CI 1.1–1.4), independently of socio‐demographic factors and symptoms. Benign diagnoses in the pre‐diagnostic year (OR = 2.01; 95% CI 1.2–3.3) and anaemia 2–5 years pre‐diagnosis (OR = 1.91; 95% CI 1.2–3.0) increased the risk of EP in women but not men. The risk was particularly high for women aged 40–59 with a recent benign diagnosis vs. none (OR = 4.41; 95% CI 1.3–14.9).

**Conclusions:**

Women have an increased risk of EP, in part due to less specific symptoms and their more frequent attribution to benign diagnoses. For women aged 40–59 years with new‐onset IBS/diverticular disease innovative diagnostic strategies are needed, which might include use of quantitative faecal haemoglobin testing (FIT) or other colorectal cancer investigations. One‐fifth of women had alarm symptoms before EP, offering opportunities for earlier diagnosis.

## 1 INTRODUCTION

Internationally, emergency colorectal cancer diagnoses range between 14% and 33% (Zhou et al., 2017), with only few studies providing separate figures for colon and rectal cancers, despite the much higher risk of emergency presentations among colon cancers (31% vs. 15% for colon and rectal cancers; Abel, Shelton, Johnson, Elliss‐Brookes, & Lyratzopoulos, 2015). In the United Kingdom, one in three colon cancers are diagnosed as an emergency (Zhou et al., 2017). Reducing emergency presentations is important as they are associated with worse 12‐month cancer survival (51% after emergency vs. more than 80% after non‐emergency colorectal cancer diagnosis; NCIN). Women, older and deprived individuals have an increased risk of emergency presentations (Abel et al., 2015; Renzi, Lyratzopoulos et al., 2016; Wallace et al., 2014; Zhou et al., 2017), with the risk for women vs. men ranging between OR = 1.2 and 1.4 (*p* < 0.05; Abel et al., 2015; Renzi, Lyratzopoulos et al., 2016). Women with colon cancer have lower 12‐month survival than men, both overall (Quaresma, Coleman, & Rachet, 2015) and across specific diagnostic routes, with women diagnosed after emergency presentation having particularly low survival (NCIN). However, evidence on the circumstances surrounding emergency presentations and on reasons for the higher risk of emergency diagnoses among women is scant.

Patient, health care and tumour factors are possible explanations (Zhou et al., 2017), including less frequent help‐seeking among some subgroups due to cancer fear, fatalism or poor cancer awareness (Robb et al., 2009), as well as delays in investigations or diagnostic difficulties due to comorbidities, benign diagnoses and atypical presentations. Risk factors might differ for men and women: for example, proximal cancers occur more frequently in women, possibly leading to gender differences in diagnostic complexity, as proximal cancers often present with non‐specific symptoms and are beyond the reach of flexible sigmoidoscopy (Holme et al., 2017). Generally, women are more frequent help seekers (Hansen, Hjertholm, & Vedsted, 2015), but no population‐based evidence exists on patterns of symptomatic presentation during the months and years before a cancer diagnosis by gender and how this might impact on emergency diagnoses.

Diagnostic pathways might also be influenced by previous diagnoses of benign conditions (Renzi, Whitaker, Winstanley, Cromme, & Wardle, 2016), such as irritable bowel syndrome (IBS) or diverticular disease, which can present with overlapping symptomatology with colon cancer (Regula, 2016), complicating symptom interpretation and differential diagnosis. Diagnostic difficulties might be particularly relevant in women, who have a higher prevalence of IBS compared with men (Lovell & Ford, 2012; Sperber et al., 2017). Overall, the incidence of colorectal cancer in patients diagnosed with IBS or diverticular disease is similar to the general population (Canavan, Card, & West, 2014; Norgaard et al., 2011; Regula, 2016). However, in the months immediately after the benign diagnosis, there is an increased risk of colon cancer (Canavan et al., 2014; Norgaard et al., 2011; Regula, 2016), especially among individuals aged <50 (Canavan et al., 2014). It is unknown whether colon cancer patients receiving a diagnosis of IBS or diverticular disease are at increased risk of an emergency rather than non‐emergency cancer diagnosis.

The present study is part of a wider project on emergency presentations based on linked cancer registry, primary and secondary care data (Renzi, Lyratzopoulos et al., 2016). We have previously shown that consultations increase markedly during the pre‐diagnostic year, independently of diagnostic route, with emergency presenters having less frequently typical alarm symptoms.

This study aimed to take the previous work further and increase our understanding on reasons for the higher risk of emergency presentations among women, in order to identify possible opportunities for earlier diagnosis overall and in women in particular. We focused on consultation patterns, signs/symptoms and benign diagnoses recorded before the colon cancer diagnosis, comparing emergency and non‐emergency presenters by gender, taking cancer sub‐sites into account. As almost half of colon cancers occur in women, reducing their risk of emergency presentations can be beneficial not only for the affected individual but also more generally for public health, in terms of overall cancer survival and reduced disruptions to hospital services.

## 2 METHODS

### 2.1 Study population and data sources

The present cohort study focused on patients with an incident colon cancer (ICD10 codes C18) diagnosed in England 2005–2010 recorded in the National Cancer Registry and individually linked to primary care data (provided by the Clinical Practice Research Datalink‐ CPRD) and secondary care data (Hospital Episode Statistics‐HES). About 6.9% of the UK population is covered by CPRD and included patients are considered to be representative of the general UK population (Herrett et al., 2015). We focused on colon cancer, rather than colorectal, given the particularly high risk of emergency presentations.

Inclusion criteria were: ages 18 years or over at cancer diagnosis, no previous cancer at any site and having at least 1 year of primary care CPRD records prior to cancer diagnosis. We excluded records not meeting the CPRD quality criteria (e.g., “up‐to‐standard” date). Patients with previous cancers were excluded as their consultation and referral patterns are likely to be different from patients with no cancer history (due to higher cancer awareness, regular follow‐up visits, and lower threshold for referrals/investigations). As expected, 6.5% of colon cancers from the cancer registry were successfully linked to active and up‐to‐standard CPRD records (*N* = 6,316 patients out of 97,937 incident colon cancers diagnosed in 2005–2010; details in Supporting Information Appendix Figure S1). After excluding patients with missing socio‐demographic or route to diagnosis information, a total of 5,745 individuals were included.

The following ethics approval was obtained: ISAC‐Protocol 08_031R; NHS Health Research Authority Confidentiality Advisory Group (PIAG 1‐05(c)/2007).

Further details on the overall project have been previously published (Renzi, Lyratzopoulos et al., 2016).

### 2.2 Study variables

The outcome of interest was emergency diagnosis, defined as a colon cancer diagnosed following presentation to Accident and Emergency, GP emergency referrals or emergency pathways for in/out‐patients, according to the Routes to Diagnosis algorithm (Elliss‐Brookes et al., 2012; NCIN). Accident and Emergency and GP emergency referrals account for 90% of emergency diagnoses and are characterised by similar 1‐year survival (NCIN). Non‐emergency diagnoses included routine GP referrals, 2‐week wait referral, inpatient/outpatient elective and screening.

The main explanatory variables were signs/symptoms recorded before the cancer diagnosis. CPRD provides patient‐level information recorded prospectively in primary care on type and timing of signs/symptoms, test results (e.g., iron‐deficiency anaemia) and referrals. Based on the literature and guidelines (Din et al., 2015; NICE Guidelines [NG12]; Sheringham, Georghiou, Chitnis, & Bardsley, 2014), we operationally defined relevant signs/symptoms that could prompt diagnostic work‐up for a possible colon cancer. Clinical experts reviewed the list and Medcodes/Readcodes for relevant symptoms (e.g., rectal bleeding, change in bowel habit, anaemia) were identified and applied to CPRD records (code‐list in AppendixS1). Clinical experts included GPs, gastroenterologist and public health specialists with a specific interest in cancer and expertise in using CPRD. In addition, colorectal cancer patients have taken part in discussing relevant signs/symptoms.

The analysis focused on primary care records referring to the pre‐diagnostic year, but earlier records, up to 5 years pre‐diagnosis, were used to examine frequency of GP consultations over time and to categorise each sign/symptom as “new” (a symptom recorded for the first time during the pre‐diagnostic year, with no prior record of the same symptom), “chronic” (recorded during the pre‐diagnostic year and at least once in previous months/years) and “past” (recorded only in the past 2–5 years, with no record in the pre‐diagnostic year). We developed this classification as we hypothesised that the effect on emergency presentation might be influenced by the timing of symptom onset and past symptom experience.

Further explanatory variables were benign intestinal conditions (irritable bowel syndrome (IBS), diverticular disease and haemorrhoids) recorded in primary care before the cancer diagnosis. We grouped IBS and diverticular disease together due to sparse data. These two conditions also have many overlapping features and often present with recurrent abdominal symptoms (Strate, Modi, Cohen, & Spiegel, 2012).

Relevant referrals for a gastro‐intestinal consultation and/or investigations (lower GI endoscopies, imaging of digestive tract, abdominal ultrasound scan, CT/MRI) recorded in CPRD during the pre‐diagnostic year were also examined. A binary variable (any relevant referral vs. none) was created. Small numbers prevented us from analysing specific referrals separately. In line with previous studies, we used three or more GP consultations with relevant symptoms as a proxy for referral delays (Lyratzopoulos, Neal, Barbiere, Rubin, & Abel, 2012).

We identified comorbidities recorded in HES using a previously developed algorithm (Maringe, Fowler, Rachet, & Luque‐Fernandez, 2017; Shack, Rachet, Williams, Northover, & Coleman, 2010). As linked HES records were available from 2003 onwards, a 2‐year pre‐diagnostic time window was chosen, in order to have the same secondary care observation period for all patients, including those diagnosed with cancer in 2005.

Cancer sub‐sites were classified into distal (left) colon (i.e., splenic flexure, descending colon, sigmoid colon; ICD C18.5–C18.7) and proximal (right) colon (i.e., caecum, appendix, ascending colon, hepatic flexure, transverse; C18.0–C18.4) (Doubeni et al., 2016; Hansen et al., 2015; Karim, Brennan, Nanji, Berry, & Booth, 2017).

Socio‐demographic characteristics included gender, age and deprivation based on the income domain of the Index of Multiple Deprivation for England.

### 2.3 Statistical analysis

We first described socio‐demographic characteristics and pre‐diagnostic signs/symptoms, benign diagnoses and comorbidities comparing emergency vs. non‐emergency presenters. Men and women were examined separately throughout. In line with previous research (Guldbrandt, Moller, Jakobsen, & Vedsted, 2017; Renzi, Lyratzopoulos et al., 2016; Sheringham et al., 2014), when analysing events occurring in the pre‐diagnostic year we excluded the 30 days pre‐diagnosis, as events occurring shortly before diagnosis might be related to the diagnostic episode itself, rather than represent opportunities for earlier diagnosis.

We used Poisson regression to examine variations in consultation rates for relevant symptoms before the cancer diagnosis by gender, age, social deprivation, comorbidities and cancer sub‐sites. Random effects were added to account for patient‐level clustering due to repeated symptomatic presentations. Consultation rates were divided into bi‐monthly and yearly time periods, in order to examine variation over time.

Mixed‐effects multivariable logistic regression was used for examining the risk of emergency presentations according to socio‐demographic characteristics, cancer sub‐site, number of consultations and type and timing of sign/symptoms, benign diagnoses and comorbidities. Random effects were added to account for clustering of patients by GP practice. We then evaluated (a) whether the effect for each sign/symptom and benign diagnoses varied for men and women, and (b) whether age modified the effect of a benign diagnosis on the risk of emergency presentation.

Finally, in order to evaluate whether effects vary by cancer sub‐site, we performed multinomial logistic regression, including all the previously mentioned variables into the model and comparing the likelihood of emergency diagnosis separately for proximal and distal cancer compared to non‐emergency colon cancer diagnosis.

Statistical analyses were performed using STATA14 software (Stata Corporation, College Station, TX, USA).

## 3 RESULTS

### 3.1 Characteristics of the study cohort and prevalence of emergency cancer diagnosis

Among the 5,745 colon cancer patients included in the study, 49% were women, with a median age of 74 years (IQR 65–82) for women and 72 for men (IQR 64–79). Our cohort had comparable demographic characteristics to colon cancer patients in the National Cancer Registry unlinked to CPRD (48% women; median age 75 [IQR 66–83] for women and 72 for men [IQR 64–80]). Proximal cancer was more frequent in women (53% vs. 45% in men, *p* < 0.001). Emergency presentations occurred in 34% of women and 30% of men, with higher risks for people from more deprived areas and the oldest and youngest age groups (Table 1). Distal cancers were associated with a lower risk of emergency presentation than proximal and unspecified colon sub‐sites.

**Table 1:** Diagnosis of colon cancer after Emergency Presentation (EP) by patients' socio‐demographic characteristics and cancer sub‐site (*N* = 5,745)

	Women			*p*‐Value^a^	Men			*p*‐Value^a^
	Non‐EP (%)	EP (%)	Total (*N*)		Non‐EP (%)	EP (%)	Total (*N*)	
	*N* = 1859	*N* = 940	*N* = 2,799		*N* = 2072	*N* = 874	*N* = 2,946	
Age (years)								
18–59	65.0	35.0	414	<0.001	68.0	32.0	431	<0.001
60–69	75.3	24.7	595		78.2	21.8	780	
70–79	72.7	27.3	868		71.4	28.6	1,010	
80+	55.4	44.6	922		61.8	38.2	725	
SES (deprivation quintile)								
1 (least deprived#)	70.4	29.6	609	0.005	71.5	28.5	708	0.057
2	66.3	33.7	602		69.9	30.1	654	
3	66.4	33.6	601		73.7	26.3	574	
4	65.9	34.1	513		68.2	31.8	529	
5 (most deprived)	58.3	41.7	350		65.2	34.8	353	
Geographic region								
North	64.2	35.8	586	0.387	69.2	30.8	708	0.877
Midlands/East England	68.5	31.5	841		70.5	29.6	863	
London	65.8	34.2	295		70.0	30.0	227	
South	66.2	33.8	1,077		71.0	29.0	1,148	
Year of CRC diagnosis								
2005–2006	63.8	36.2	889	0.126	67.1	32.9	902	0.034
2007–2008	67.3	32.7	924		72.2	27.8	960	
2009–2010	68.0	32.1	986		71.4	28.6	1,084	
Cancer sub‐site								
Colon proximal	66.8	33.2	1,477	0.043	69.2	30.8	1,324	<0.001
Colon distal	67.8	32.2	1,010		73.8	26.2	1,329	
Colon unspecified	60.3	39.7	312		59.7	40.3	293	

Notes

Distal colon: splenic flexure, descending colon, sigmoid colon; Proximal colon: transverse and ascending colon.

a Chi‐square test comparing emergency vs. non‐emergency presenters.

### 3.2 Consultation pattern before a colon cancer diagnosis by gender and cancer sub‐site

**Figure 1:**
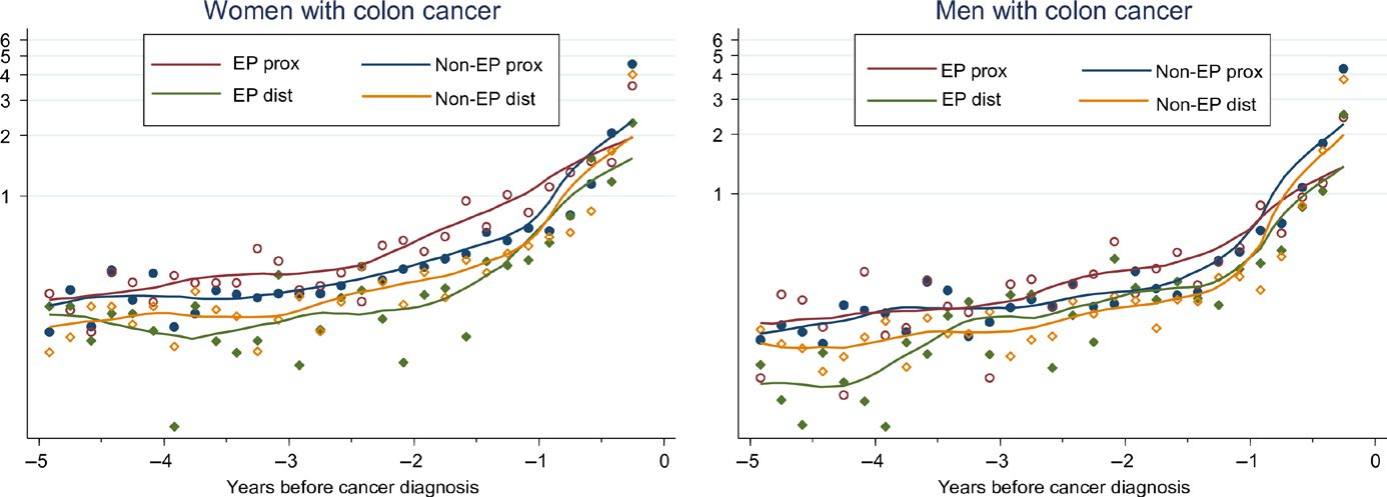
Consultation rates with relevant symptoms for men and women with proximal or distal colon cancer diagnosed following an emergency presentation (EP) and non‐emergency presentation (non‐EP). Note: Observed data points and fitted local polynomial regression lines on logarithmic scale

Consultation rates with relevant signs/symptoms (rectal bleeding, change in bowel habit, anaemia, abdominal pain, constipation) started increasing during the 1–2 years pre‐cancer diagnosis, independently of gender, diagnostic route and cancer sub‐sites (Figure 1). A particularly sharp increase was observed in the pre‐diagnostic year, with the important exception of women with proximal cancer diagnosed as an emergency, whose increase started 2 years pre‐diagnosis. Patients with proximal cancer had higher consultation rates than those with distal cancers, with consultations increasing earlier for emergency presenters, particularly among women.

Consultation rates with relevant symptoms were significantly higher for women compared to men, taking age, deprivation and cancer sub‐site into account (Supporting Information Appendix Figure S2). Consultation rates were also higher for older patients and those in more deprived areas, while distal cancers had lower consultation rates compared to proximal cancers. Results were similar repeating the analyses including also diagnostic route and comorbidity in the multivariable model (data not shown).

### 3.3 Symptoms and benign diagnoses before emergency and non‐emergency presentation by gender

#### 3.3.1 Relevant symptoms

The proportion of patients with at least one consultation for relevant symptoms in the pre‐diagnostic year was higher among women than men (60% vs. 55%, *p* < 0.001), with women also having more frequently 3 + consultations with relevant symptoms (17% vs. 15%, *p* < 0.001; Table 2). Emergency presenters of either sex had less frequently relevant symptoms compared to non‐emergency presenters. However, among women diagnosed as an emergency, 20% had alarm symptoms (anaemia, rectal bleeding, change in bowel habit) during the pre‐diagnostic year, vs. 15% among men (*p* = 0.002).

**Table 2:** GP consultations, symptoms and benign diagnoses among patients diagnosed with colon cancer following emergency presentation (EP) and Non‐emergency presentation (non‐EP)

	Women				Men				Women vs. Men EP only	Women vs. Men total sample
	Non‐EP (%)	EP (%)	Total (%)	*p*‐Value^a^	Non‐EP (%)	EP (%)	Total (%)	*p*‐Value^a^	*p*‐Value^b^	*p*‐Value^c^
	*N* = 1859	*N* = 940	*N* = 2,799		*N* = 2072	*N* = 874	*N* = 2,946			
No. of consultations with relevant symptoms between 2–12 months pre‐diagnosis										
0 consultations	34.1	50.6	39.7	<0.001	39.8	57.6	45.0	<0.001	0.012	<0.001
1–2 consultations	47.8	33.8	43.1		44.7	29.6	40.2			
3+ consultations	18.1	15.5	17.3		15.5	12.8	14.7			
At least one alarm symptom (anaemia, rectal bleeding, change in bowel habits)										
2–12 months pre‐diagnosis	37.7	20.1	31.8	<0.001	34.5	14.5	28.6	<0.001	0.002	0.009
12–23 months	7.4	8.3	7.7	0.413	5.3	5.3	5.3	0.960	0.010	<0.001
24–36 months	4.5	5.3	4.8	0.316	3.1	4.4	3.5	0.102	0.336	0.017
Specific symptoms										
Change in bowel habit										
New onset	6.7	2.0	5.1	<0.001	7.0	2.4	5.6	<0.001	0.675	0.347
Chronic	0.1	0.1	0.1		0.1	0.1	0.1			
Past	1.6	1.5	1.6		1.2	0.9	1.1			
Rectal bleeding										
New onset	10.1	3.3	7.8	<0.001	10.3	2.9	8.1	<0.001	0.427	0.940
Chronic	1.2	0.7	1.0		1.1	0.5	0.9			
Past	3.2	3.2	3.2		3.5	2.2	3.1			
Anaemia										
New onset	17.5	11.7	15.6	<0.001	15.8	7.7	13.4	<0.001	<0.001	<0.001
Chronic	4.1	3.4	3.9		2.5	2.0	2.3			
Past	4.6	7.8	5.7		3.4	5.4	4.0			
Abdominal pain										
New onset	15.1	16.6	15.6	0.781	14.7	14.3	14.6	0.987	0.024	<0.001
Chronic	6.8	6.7	6.8		4.2	4.4	4.2			
Past	10.4	10.0	10.3		8.5	8.6	8.5			
Constipation										
New onset	5.6	6.2	5.8	0.023	4.9	5.8	5.2	0.060	0.021	0.014
Chronic	1.6	3.3	2.1		1.5	1.1	1.4			
Past	6.6	6.6	6.6		4.6	6.8	5.3			
Diarrhoea										
New onset	6.0	6.8	6.3	0.390	6.0	6.0	6.0	0.942	0.028	<0.001
Chronic	2.5	1.6	2.2		0.9	1.0	0.9			
Past	8.5	8.9	8.6		5.3	5.7	5.4			
Fatigue										
New onset	3.9	3.4	3.7	0.160	3.2	3.1	3.2	0.596	<0.001	<0.001
Chronic	1.8	0.9	1.5		0.7	0.3	0.6			
Past	7.8	8.8	8.2		4.8	4.1	4.6			
Weight loss										
New onset	1.9	2.0	2.0	0.962	2.5	2.9	2.6	0.796	0.574	0.061
Chronic	0.1	0.1	0.1		0.2	0.1	0.2			
Past	2.0	2.1	2.1		1.3	1.6	1.4			
Benign GI diagnosis recorded between 2–12 months pre‐diagnosis										
IBS or Diverticular disease^d^										
New onset	5.1	6.0	5.4	0.678	3.4	2.3	3.1	0.258	0.000	0.000
Chronic	0.8	0.9	0.8		0.5	0.2	0.4			
Past	4.6	5.2	4.8		2.1	2.3	2.2			
Haemorrhoids										
New onset	2.0	0.7	1.6	0.013	2.0	1.1	1.8	0.344	0.421	0.905
Chronic	0.5	0.2	0.4		0.5	0.3	0.5			
Past	3.2	2.0	2.8		2.8	3.0	2.8			
3+ Pre‐referral consultations for relevant symptoms pre‐diagnosis (only patients with referral: 687 women and 674 men)										
	14.2	21.7	15.6	0.033	12.7	21.0	14.0	0.027	0.897	0.397
Comorbidities recorded in HES between 0–24 months pre‐diagnosis										
0	77.6	63.3	72.8	<0.001	74.5	58.1	69.6	<0.001	0.038	0.003
1–2	19.4	30.0	22.9		20.8	32.7	24.3			
3+	3.1	6.7	4.3		4.7	9.2	6.0			

Notes

New onset: symptom recorded for the first time during the pre‐diagnostic year with no prior record of the same symptom; Chronic: recorded both during the pre‐diagnostic year and in previous years; Past: recorded only in the past 2–5 years, with no record in the pre‐diagnostic year.

a Chi‐square test comparing EP vs. non‐EP.

b Chi‐square test comparing women EP vs. men EP.

c Chi‐square test comparing women vs. men overall (including EP and non‐EP).

d Irritable bowel syndrome (IBS) and diverticular disease were grouped together due to sparse data (diverticular disease *n* = 183 and IBS *n* = 98).

Past anaemia 2–5 years pre‐diagnosis was more frequent among emergency presenters, particularly for women. Anaemia, abdominal pain, constipation and fatigue were more frequently recorded in women than men.

#### 3.3.2 Benign diagnoses

Women more often had a record of a benign diagnosis (IBS/diverticular disease) in the pre‐diagnostic year: 6% vs. 2% among women and men diagnosed as an emergency (*p* < 0.001) (Table 2). Similarly, in the subgroup of people with abdominal symptoms (change in bowel habit, abdominal pain, constipation or diarrhoea, *n* = 2046), there was a greater probability of benign diagnoses during the pre‐diagnostic year for women (9.5%) vs. men (5.2%) (*p* < 0.001). Further restricting this analysis to persons with abdominal symptoms who were diagnosed as emergencies (*n* = 574), 12.4% of women vs. 6% of men received a benign diagnosis (*p* = 0.002) (data not shown in table). This gender disparity in benign diagnoses was also observed when restricting the analysis to emergency presenters with alarm symptoms (change in bowel habit, rectal bleeding or anaemia): 9.5% vs. 2.4% (*p* = 0.01) of women and men had received a recent benign diagnosis.

### 3.4 Multivariable analysis examining factors associated with emergency presentation by gender and cancer sub‐site

At multivariable analysis, emergency presentations were more likely in women (OR = 1.20; 95% CI 1.1–1.4), as well as among the oldest and youngest age groups and the most deprived, independently of symptoms, number of consultations and cancer sub‐site (Table 3). Multiple pre‐referral consultations with relevant symptoms (OR = 1.25; 95% CI 1.1–1.6) and comorbidities also increased the risk of emergency presentations, while new‐onset alarm symptoms decreased the risk.

**Table 3:** Mixed‐effects logistic regression Odds Ratios (OR) for colon cancers diagnosed after Emergency Presentation (EP) vs. non‐EP, taking socio‐demographic characteristics, GP consultations and clinical history into account (*N* = 5,745)

	Adjusted ORs for both genders			
	OR	95% CI		*p*‐Value
Gender				
Men	1			
Women	1.20	1.06	1.35	0.005
Age (years)				
18–59	1.83	1.50	2.24	<0.001
60–69	1			
70–79	1.32	1.11	1.57	0.002
80+	2.23	1.87	2.67	<0.001
SES (deprivation quintile)				
1 (least deprived#)	1			
2	1.17	0.98	1.40	0.091
3	1.03	0.86	1.24	0.758
4	1.14	0.94	1.37	0.186
5 (most deprived)	1.39	1.13	1.72	0.002
Year of diagnosis				
2005–2006	1			
2007–2008	0.79	0.68	0.92	0.002
2009–2010	0.78	0.67	0.90	0.001
Cancer sub‐site				
Colon proximal	1			
Colon distal	0.93	0.82	1.07	0.323
Colon unspecified	1.28	1.05	1.56	0.016
No. visits during 2–12 months pre‐diagnosis	0.99	0.98	0.99	<0.001
Change in bowel habit				
Never	1			
New onset	0.30	0.21	0.43	<0.001
Chronic/past	0.70	0.41	1.19	0.184
Rectal bleeding				
Never	1			
New onset	0.26	0.19	0.35	<0.001
Chronic/past	0.62	0.45	0.87	0.005
Anaemia				
Never	1			
Chronic	0.60	0.41	0.87	0.007
New onset	0.44	0.36	0.53	<0.001
Past	1.20	0.91	1.58	0.201
Abdominal pain				
Never	1			
Chronic	0.98	0.73	1.31	0.880
New onset	0.99	0.82	1.19	0.914
Past	1.06	0.85	1.32	0.604
Constipation				
Never	1			
Chronic	1.40	0.89	2.20	0.145
New onset	1.13	0.86	1.48	0.392
Past	1.13	0.87	1.47	0.352
Diarrhoea				
Never	1			
Chronic	0.78	0.47	1.32	0.361
New onset	1.04	0.81	1.35	0.743
Past	1.01	0.79	1.29	0.928
Fatigue				
Never	1			
Chronic	0.43	0.21	0.86	0.017
New onset	0.92	0.66	1.29	0.632
Past	0.95	0.73	1.22	0.668
Weight loss				
Never	1			
New onset	1.01	0.68	1.50	0.954
Chronic/past	1.10	0.69	1.74	0.695
IBS or Diverticular disease				
Never	1			
New onset	1.06	0.78	1.45	0.696
Chronic/past	1.19	0.87	1.63	0.278
Haemorrhoids				
Never	1			
New onset	0.49	0.27	0.90	0.022
Chronic/past	0.84	0.58	1.22	0.361
3+ Pre‐referral consultations with relevant symptoms 2–12 months pre‐diagnosis				
0–2	1			
3+	1.25	1.06	1.56	0.048
Comorbidities recorded in HES between 0–24 months pre‐diagnosis				
0	1			
1+	2.15	1.87	2.48	<0.001

Notes

New onset: symptom recorded for the first time during the pre‐diagnostic year with no prior record of the same symptom; Chronic: recorded both during the pre‐diagnostic year and in previous years; Past: recorded only in the past 2–5 years, with no record in the pre‐diagnostic year.

**Figure 2:**
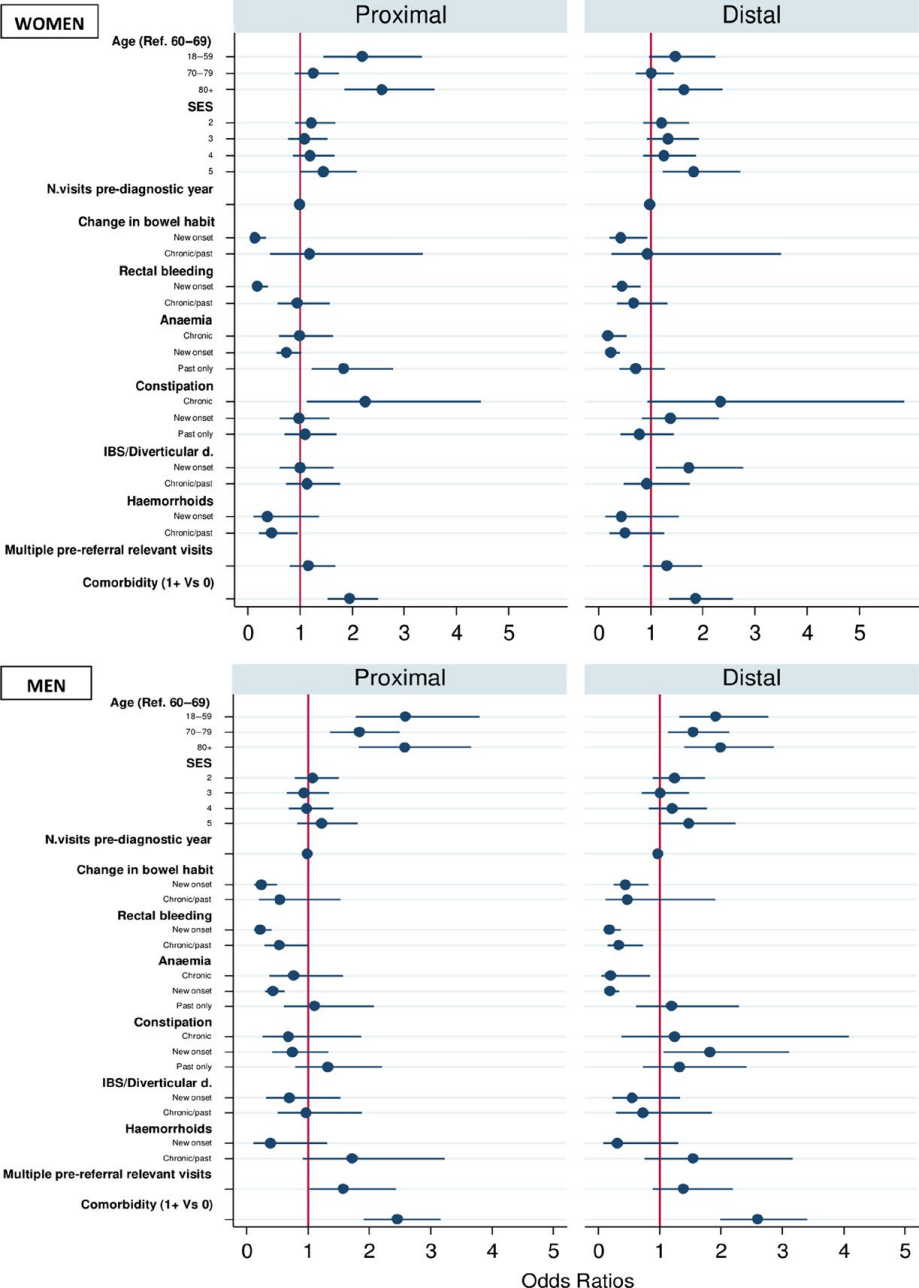
Odds Ratios for women and men diagnosed with proximal or distal colon cancer after Emergency Presentation (EP) compared to non‐EP. Note: Multinomial logistic regression taking socio‐demographic characteristics, GP consultations and clinical history into account

Among women, a recent benign diagnosis (OR = 2.01; 95% CI 1.2–3.3) and a past history of anaemia 2–5 years pre‐diagnosis (OR = 1.91; 95% CI 1.2–3.0) increased the risk of emergency presentation in patients with distal and proximal cancers, respectively (Figure 2). No such association was observed for past/chronic benign diagnoses and no association was apparent between benign diagnoses and emergency presentations in men. Results were similar when analysing each cancer sub‐site and gender separately (Supporting Information Appendix Tables S1–S2), but without reaching statistical significance due to sparse data in stratified analyses.

### 3.5 Benign diagnoses and effect on emergency presentations among women stratified by age

The prevalence of a benign diagnosis among women with colon cancer diagnosed as emergencies was particularly high among 40–49‐year‐olds (18.4%), while it was 8.8% in 50–59‐year‐olds and lower in all other age groups (4.5%–6.8%; data not shown in table). Concordantly, there was statistical evidence of effect modification for a recent benign diagnosis on the risk of emergency presentation by age (likelihood ratio test *p* = 0.013).

Multivariable logistic regression stratified by age and controlling for deprivation, cancer sub‐site and symptoms highlighted how the risk of emergency presentation was particularly high for 40–59‐year‐old women with a recent benign diagnosis compared to those without a benign diagnosis (OR = 4.41; 95% CI 1.3–14.9). There was no significant effect of a benign diagnosis on all other groups (Table 4).

**Table 4:** Odds Ratios (OR) for the association between a recent benign diagnosis (IBS/diverticular disease) and emergency presentations among women only, stratified by age

	Adjusted ORs for women only			
	OR	95% CI		*p*‐Value
Age (years)				
40–59	4.41	1.3	14.9	0.017
60–69	1.43	0.6	3.7	0.464
70+	1.29	0.8	2.1	0.322

Mixed effects binary logistic regression ORs estimated in separate models for each age group, controlling for sociodemographic factors, cancer sub‐site and symptoms.

## 4 DISCUSSION

### 4.1 Main findings

The study provides population‐based evidence on factors associated with emergency colon cancer diagnosis in women and men, highlighting possible opportunities for earlier diagnosis. Consultation rates with relevant symptom pre‐cancer diagnosis were higher in women than men and increased substantially in the pre‐diagnostic year; the increase in relevant consultations occurred earlier in women with proximal colon cancer, who were also at increased risk of emergency diagnosis. Women with abdominal symptoms in the pre‐diagnostic year were twice as likely to be diagnosed with a benign condition (IBS or diverticular disease) compared to men with similar symptoms. A new‐onset benign condition and a past history of anaemia 2–5 years pre‐diagnosis were associated with emergency presentations in women, but not in men. A particularly high risk of emergency presentations was observed among women aged 40–59 with a new‐onset benign diagnosis, highlighting the need for innovative diagnostic strategies for this patient group. These may include use of quantitative faecal haemoglobin testing (FIT) or other colorectal cancer investigations.

### 4.2 Comparison with previous literature

IBS, affecting about 8% of the general population in Western countries and occurring more frequently in women (Lovell & Ford, 2012; Sperber et al., 2017) can present with abdominal pain, altered bowel habit and bloating in the absence of detectable organic disease. According to guidelines, the diagnosis is based on clinical criteria (Rome IV criteria) without the need for extensive investigations to exclude other conditions (Canavan et al., 2014; Ford, Lacy, & Talley, 2017; Mearin et al., 2016; Moayyedi et al., 2017; NICE guidelines [NG12]; Spiegel, Farid, Esrailian, Talley, & Chang, 2010), as the diagnostic yield is low in the absence of alarm signs/symptoms (Chey et al., 2010). IBS does not increase the risk of colon cancer overall, with IBS patients having similar cancer incidence as the general population (Canavan et al., 2014; Norgaard et al., 2011). However, in the first 6 months after an IBS diagnosis colorectal cancer incidence is 4–41 times higher than in controls (Canavan et al., 2014), with patients younger than 50 having the highest risk. This might be due to symptoms being initially attributed to the benign diagnosis, while subsequent investigations revealed the underlying cancer. Contrary to guidelines, many gastroenterologists and primary care doctors believe IBS is a diagnosis of exclusion and refer patient for tests to exclude serious organic conditions (Spiegel et al., 2010). Spiegel et al. (2010) also highlighted that often doctors make an “internal diagnosis” when visiting patients, without being willing to “externalise” the diagnosis to the patient; less than half of doctors were willing to communicate the diagnosis without first performing additional testing. This suggests that the relatively low IBS prevalence in our study is an underestimation of cases where the doctor made an “internal diagnosis.”

Similar to IBS, diverticular disease is a chronic disorder which often presents with recurrent abdominal symptoms (Strate et al., 2012). While not increasing the risk of colon cancer overall, during the first year of a diverticular disease diagnosis there is a strong association with colon cancer, probably due to misclassification and difficulties with differential diagnosis (Regula, 2016). Both IBS and diverticular disease might provide “alternative explanations” to cancer. This is in line with a study (Mounce, Price, Valderas, & Hamilton, 2017) reporting how comorbidities providing “alternative” explanations (including IBS and diverticular diseases) are associated with longer diagnostic intervals for colorectal cancer. Our study shows that for patients with an underlying colon cancer, receiving an IBS or diverticular disease diagnosis can be associated with an increased risk of emergency presentation. Patients might feel over‐reassured or under‐supported (Renzi, Whitaker et al., 2016) after a benign diagnosis and thus not return to their doctor even if symptoms worsen, or there might be delays in referrals for investigations.

Further work is necessary to examine these possible mechanisms, as well as examining whether benign diagnoses were supported by previous investigations. Using data from electronic health records (as in our study) can reveal patient groups at greater risk of emergency presentation, there remain however questions about the exact circumstances that could allow for such presentations to be prevented. In that respect, qualitative record review studies can be very important. For example, in a study reviewing the clinical records of Scottish patients (Murchie et al., 2017) 30% of emergency presenters previously seen by their GP with relevant symptoms had an emergency diagnosis while awaiting a secondary care appointment; 19% experienced a genuine missed opportunity for earlier investigation (with missed opportunities occurring more frequently in women than men); while only a small minority of patients had refused or did not attend follow‐up appointments or investigations. Similar approaches have also been employed in US healthcare settings (Singh et al., 2009). Complementing such detailed case note reviews with population‐based epidemiological studies like ours is important to provide a more comprehensive picture and inform the development of strategies for reducing emergency presentation at population level. By identifying subgroups of the population at higher risk, epidemiological studies can also help priorities further in‐depth case note review studies.

In our study we have shown that multiple pre‐referral consultations were associated with emergency presentations, indicating how generally prompt specialist referrals can reduce emergency diagnoses. Women are known to have more frequent consultations (Abel et al., 2017; Hansen et al., 2015), longer diagnostic intervals (Din et al., 2015) and higher risk of three or more consultations before specialist referrals, particularly in the context of urinary tract cancer (Cohn, Vekhter, Lyttle, Steinberg, & Large, 2014; Lyratzopoulos, Abel, McPhail, Neal, & Rubin, 2013). It has been suggested that general practitioners interpret symptoms, such as haematuria, differently in women compared to men, with possible misattribution of symptoms to benign causes, early in the diagnostic process (Cohn et al., 2014; Lyratzopoulos et al., 2013). This might be a consequence of positive predictive values of various possible cancer symptoms being lower in women than in men (Hamilton et al., 2009; Jones, Latinovic, Charlton, & Gulliford, 2007). Differential diagnosis in women can be particularly complicated as recurrent abdominal pain and/or anaemia can sometimes be related to gynaecological conditions with a risk of over‐reassurance or false reassurance. Specifically designed quantitative and qualitative studies would be needed to explore these issues further. A greater understanding of the interplay between gender, age, benign diagnoses, chronic morbidities and symptomatic presentations is necessary in order to optimise diagnostic approaches and guidelines for higher risk groups. For example, new‐onset IBS in middle‐aged women is an indication for CA125 testing according to NICE guidelines (NICE guidelines [NG12]), yet new‐onset IBS is not currently considered a clear indication for quantitative faecal haemoglobin testing (FIT).

Anaemia is a well‐known symptom associated with colon cancer (Hamilton et al., 2009) and our findings have highlighted that while new‐onset alarm symptoms, including new‐onset anaemia, decreased the risk of emergency presentation, a long‐standing history of anaemia in women can lead to emergency presentations. Previous research also suggested that anaemia was a frequent missed diagnostic opportunity in colorectal cancer (Singh et al., 2009).

### 4.3 Limitations

The study does not reflect the prevalence of symptoms and benign diagnoses in the general population, nor does it aim to evaluate the predictive value of symptoms comparing cancer patients with the general population, as performed in other studies (Hamilton et al., 2009). Rather, our study focused on identifying factors associated with emergency presentations and opportunities for preventing them among cancer patients. We relied on clinical records of symptoms/signs which do not necessarily represent all symptoms experienced by patients. Despite the likely underestimation of sign/symptoms and diagnoses (Price, Stapley, Shephard, Barraclough, & Hamilton, 2016), we have no reason to expect differential recording by emergency presentation status, as information was prospectively recorded during the months and years before the cancer diagnosis.

The clinical management of patients with anaemia 2–5 years pre‐diagnosis merits further examination. Danish studies showed an increase in prescriptions prior to a colon cancer diagnosis (Pottegard & Hallas, 2017). Similar to our findings, this suggests that symptoms are attributed to benign diagnoses in some patients. Further work is needed exploring the effect of gynaecological and other coexisting conditions on the risk of delayed cancer diagnosis and emergency presentations.

Our study will need to be extended to more recent years. However, even though emergency presentations for colorectal cancer have decreased in England between 2006 and 2010 (from 27% to 23%), they have remained around 23% since 2010 (NCIN). Specific data for colon cancer is limited to 2006–2010 (31% diagnosed as an emergency; Abel et al., 2015). It is noteworthy that inequalities in emergency presentations (Abel et al., 2015) and cancer survival (Ellis, Coleman, & Rachet, 2012) are still relevant.

### 4.4 Implications for research and practice

Our population‐based study suggests that there are opportunities for earlier colon cancer diagnosis and for reducing emergency presentations in some patients. The findings are unlikely to be simply explained by poor clinical practice of some doctors, considering that we analysed prospectively collected data from 343 GP practices in England (average of 3 colon cancers per practice/year), taking possible clustering by practice into account. Further quantitative and qualitative studies are needed in order to gain additional insights into the doctor–patient interactions preceding the emergency cancer diagnoses and the clinical management of symptomatic patients, including an analysis of prescriptions, referrals and type and timing of investigations performed before an emergency cancer diagnosis. Considering that emergency presentations occur due to a complex interplay between patient, cancer and healthcare‐related factors (Holme et al., 2017; Lyratzopoulos, Wardle, & Rubin, 2014; Murchie et al., 2017; Zhou et al., 2017), multifaceted system‐wide approaches are probably needed, together with innovations in diagnostic technology and optimising screening. The majority of colorectal cancers are diagnosed after symptoms have developed (Goodyear et al., 2008), thus earlier diagnosis in symptomatic patients remains crucial. Attention is needed not only for people with typical alarm symptoms, but also for those repeatedly presenting with lower risk symptoms, especially in the case of women. Organizational innovations in primary care can help improve the diagnosis and management of complex cases (BMA, 2016; Hobbs et al., 2016; Rimmer, 2017). Greater integration with specialists and multi‐disciplinary diagnostic centres (Independent Cancer Taskforce, 2015) can also facilitate early diagnosis.

Specific attention is warranted for women aged 40–59 years with a recent diagnosis of IBS or diverticular disease, as this age group is characterised by an increase in colon cancer incidence, paralleled by a decrease in new IBS onset. It is noteworthy that for women aged 50 and over who have been diagnosed with IBS for the first time in the last year, NICE guidelines in the UK recommend investigations for ovarian cancer, as IBS rarely starts at this age (NICE guidelines [NG12]), while no specific recommendation is made regarding the possibility of colon cancer in these patients, unless typical alarm symptoms are present. According to international experts, a colonoscopy is indicated for all patients aged 50 years and over with symptoms such as diarrhoea and mixed bowel habit (Ford et al., 2017; Moayyedi et al., 2017). Relatedly, the American Gastroenterology Association recently recommended excluding colon cancer with modern techniques and colonoscopy after the first episode of diverticulitis (Regula, 2016).

Our findings suggest that women aged 40–59 with symptoms compatible with a recent onset IBS or diverticular disease are at increased risk of emergency diagnosis and appropriate strategies will need to be developed to address this. In addition to safety netting and specialist advice, quantitative FIT could be considered. According to recent research (Hogberg, Karling, Rutegard, & Lilja, 2017; Mowat et al., 2016) and NICE guidelines (NICE guidelines [NG12]), quantitative FIT can be useful for patients presenting with abdominal symptoms in primary care in order to identify those who might benefit from further investigations.

With one in three colon cancers diagnosed as an emergency and considering that 12‐month survival for patients with an emergency colorectal cancer diagnosis is 51%, compared to more than 80% for non‐emergency routes (NCIN), it is important to develop innovative diagnostic strategies. Reducing emergency presentations and addressing inequalities will help to improve patient experience, quality of care and cancer survival.

## CONFLICT OF INTEREST

The authors have no conflict of interest to declare.
