# Supplementary figures and images for: Opportunities for reducing emergency diagnoses of colon cancer in women and men: A data‐linkage study on pre‐diagnostic symptomatic presentations and benign diagnoses
Source: Eur J Cancer Care (Engl). 2019 Feb 8;28(2):e13000. doi: 10.1111/ecc.13000 (PMC6492167; doi:10.1111/ecc.13000)

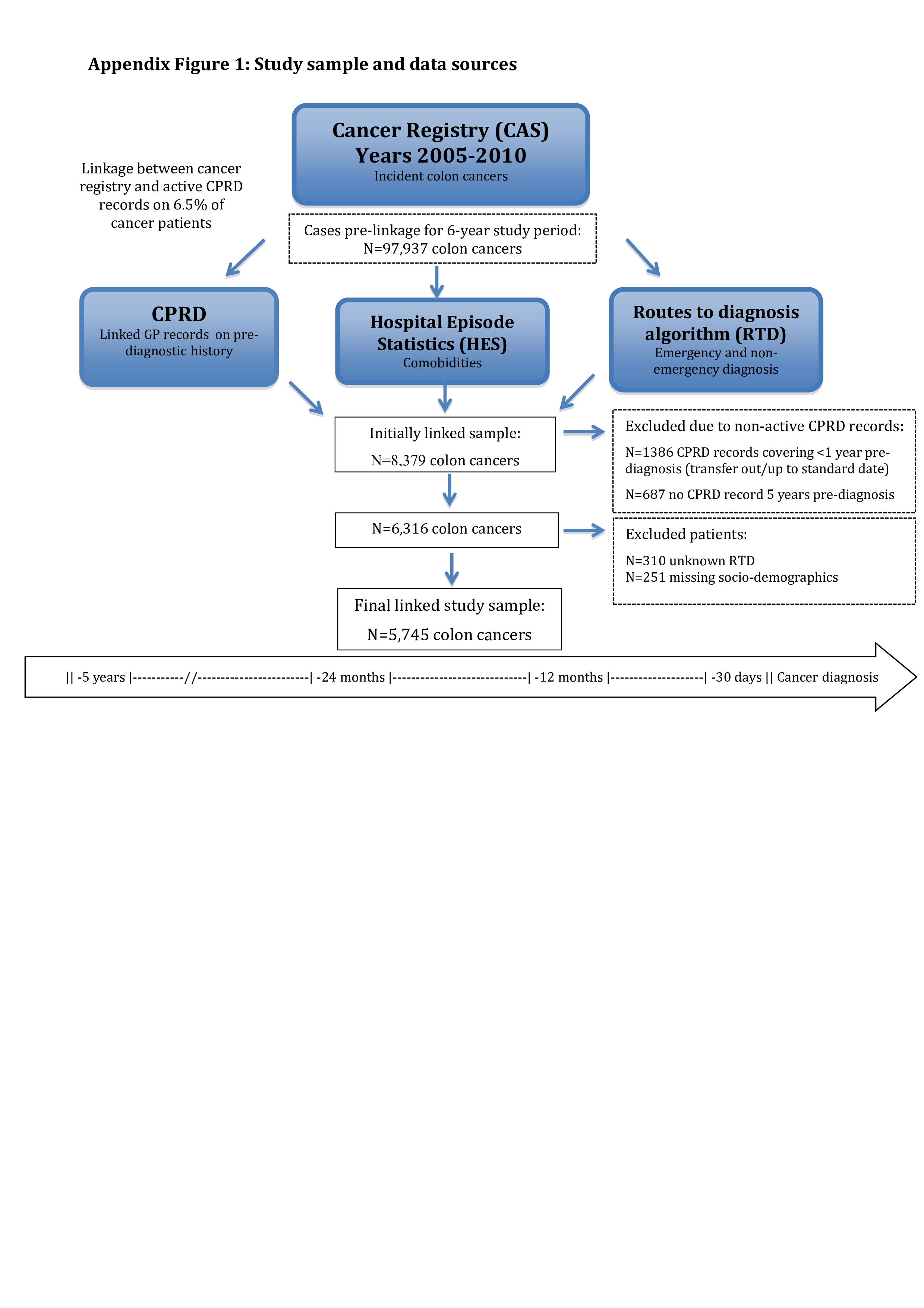

Supplement: Supplementary file 1 [file ECC-28-na-s001.tif]

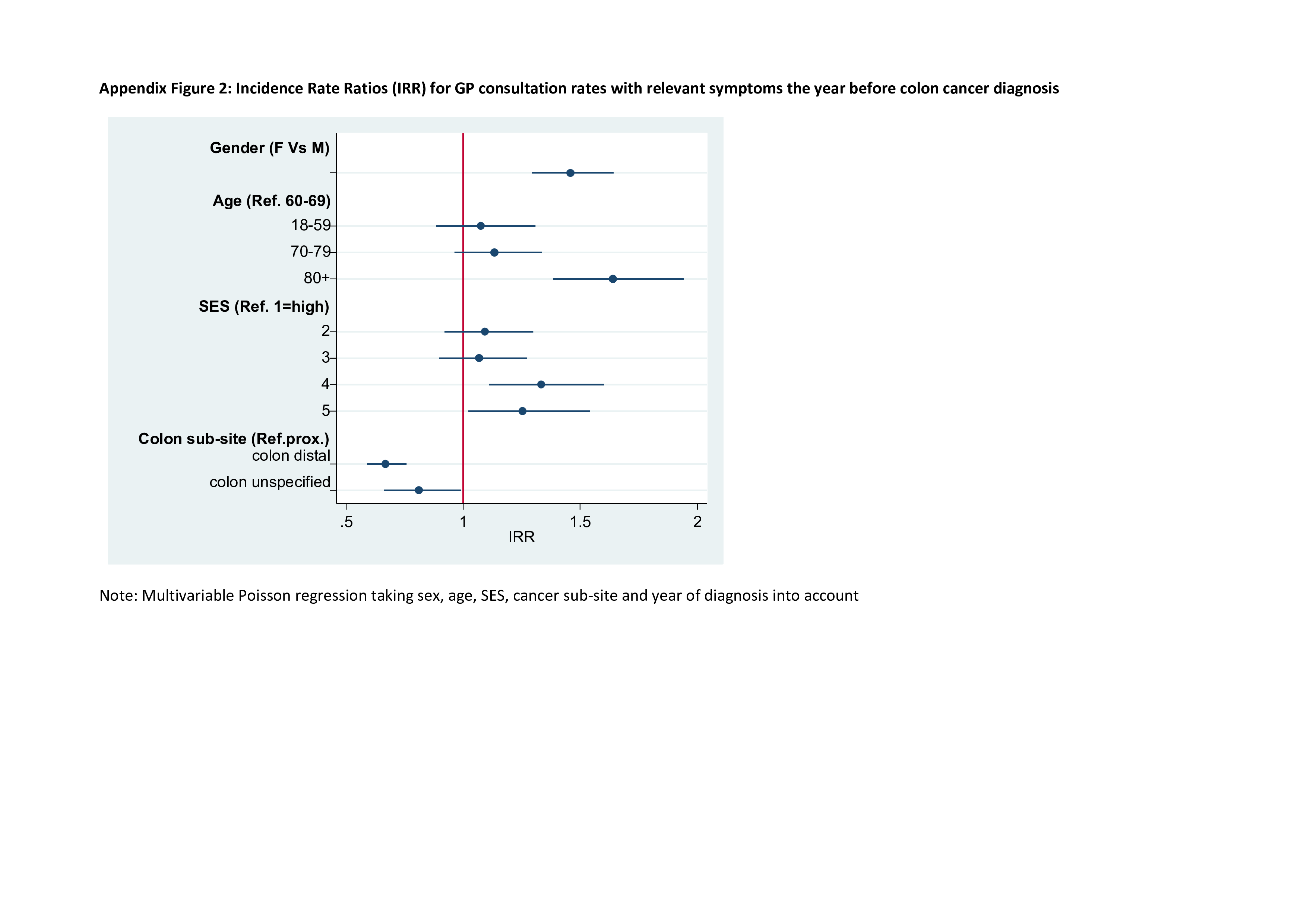

Supplement: Supplementary file 2 [file ECC-28-na-s002.tif]
