# Supplementary material for: Opportunities for reducing emergency diagnoses of colon cancer in women and men: A data‐linkage study on pre‐diagnostic symptomatic presentations and benign diagnoses
Source: Eur J Cancer Care (Engl). 2019 Feb 8;28(2):e13000. doi: 10.1111/ecc.13000 (PMC6492167; doi:10.1111/ecc.13000)
Supplement: Supplementary file 4 [file ECC-28-na-s004.docx]

| **Appendix Codelist** | |  |
| --- | --- | --- |
| **Readcodes** | **Medcodes** | **Description** |
| 22A8.00 | 37937 | Weight loss from baseline weight |
| R032.00 | 3647 | [D]Abnormal loss of weight |
| 1625.11 | 5812 | Abnormal weight loss - symptom |
| 1D1A.00 | 12398 | Complaining of weight loss |
| 1625.00 | 4663 | Abnormal weight loss |
| 1627.00 | 102563 | Unintentional weight loss |
| 19DZ.00 | 41896 | Tenesmus NOS |
| 19D2.00 | 38499 | Tenesmus present |
| R07z000 | 15756 | [D]Tenesmus |
| 19D..00 | 14948 | Tenesmus |
| 19D..11 | 6541 | Tenesmus symptom |
| 4762.11 | 14256 | Blood in faeces |
| J681.11 | 2873 | Blood in stool |
| J573011 | 621 | Rectal bleeding |
| 19E6.11 | 6151 | Blood in faeces symptom |
| 19E6.00 | 5462 | Blood in faeces |
| J573000 | 6574 | Rectal haemorrhage |
| 196B.00 | 11718 | Painful rectal bleeding |
| 196C.00 | 11698 | Painless rectal bleeding |
| 4762.00 | 44004 | Faeces: fresh blood present |
| J573z00 | 46479 | Haemorrhage of rectum and anus NOS |
| 14CA.11 | 17027 | H/O: GI Bleed |
| J681.13 | 20859 | Blood in stools altered |
| 19ED.00 | 9968 | Blood on toilet paper |
| J573.11 | 3872 | Bleeding PR |
| J573012 | 6554 | PRB - Rectal bleeding |
| J681.12 | 27862 | Altered blood in stools |
| J573.00 | 19271 | Haemorrhage of rectum and anus |
| Jyu5200 | 70200 | [X]Other and unspecified intestinal obstruction |
| J50zz12 | 21568 | Large bowel obstruction NOS |
| J50z.00 | 935 | Intestinal obstruction NOS |
| J50z500 | 1307 | Subacute intestinal obstructuon |
| J50zz13 | 1347 | Small bowel obstruction NOS |
| J50y.11 | 18240 | Intestinal adhesions with obstruction |
| J50z400 | 28322 | Acute intestinal obstruction |
| J50y000 | 29118 | Intestinal adhesions with obstruction |
| J50y.00 | 42547 | Other intestinal obstruction |
| J504.00 | 100679 | Distal intestinal obstruction syndrome |
| J50zz00 | 1544 | Intestinal obstruction NOS |
| J50zz11 | 18789 | Colonic obstruction NOS |
| J50zz15 | 1221 | Bowel obstruction |
| J50..00 | 20471 | Intestinal obstruction without mention of hernia |
| SP14400 | 23478 | Intestinal obstruction as a complication of care NOS |
| J50y300 | 60824 | Intestinal mural thickening with obstruction |
| J50zz14 | 21648 | Subacute intestinal obstruction NOS |
| J50yz00 | 33759 | Other intestinal obstruction NOS |
| 25N2.00 | 69694 | O/E -abd.mass-lower border def |
| 25J1.00 | 25588 | O/E - abd. mass not palpated |
| 25J2.00 | 57745 | O/E - abd. mass < 1 quadrant |
| 25L..00 | 20387 | O/E - abdominal mass shape |
| 25L2.00 | 65538 | O/E -abd.mass -irregular shape |
| 25LZ.00 | 43306 | O/E - abd. mass shape NOS |
| 25J5.00 | 63484 | O/E - abd. mass fills abdomen |
| 25J4.00 | 42172 | O/E - abd. mass fills half abd |
| R093z00 | 16370 | [D]Swelling, mass or lump within abdomen or pelvis NOS |
| 7H2C500 | 34238 | Biopsy of abdominal mass |
| 25L1.00 | 53699 | O/E - abd. mass -regular shape |
| 25K..00 | 21301 | O/E-abdominal mass consistency |
| 25J7.00 | 20827 | Right iliac fossa mass |
| R093200 | 7073 | [D]Abdominal lump |
| 25KZ.00 | 59025 | O/E - abd.mass consistency NOS |
| 25N1.00 | 67090 | O/E -abd.mass-upper border def |
| 25K4.00 | 17246 | O/E - abdominal mass-pulsatile |
| 25R2.00 | 74000 | O/E - tympany over abd. mass |
| 25K1.00 | 21686 | O/E - abdominal mass - soft |
| R093700 | 24034 | [D]Umbilical mass |
| 25M2.00 | 72512 | O/E - abd.mass still with resp |
| 25Q3.00 | 1987 | O/E - PR - rectal mass |
| 25J8.00 | 21575 | O/E left lower abdominal mass |
| 25M1.00 | 68939 | O/E - abd.mass moves with resp |
| R093000 | 4800 | [D]Abdominal swelling |
| 25J..00 | 8731 | O/E - abdominal mass palpated |
| 25JZ.00 | 56675 | O/E - abd. mass palpated NOS |
| 25R3.00 | 64153 | O/E - dullness over abd. mass |
| R093100 | 3015 | [D]Abdominal mass |
| 25MZ.00 | 98951 | O/E - abd.mass + respn. NOS |
| 25N..00 | 99958 | O/E - abd.mass -border defined |
| 25M..00 | 59578 | O/E - abd.mass movt.with resp. |
| 25A2.00 | 30120 | O/E - uniform abd. swelling |
| 25NZ.00 | 68388 | O/E -abd.mass -border def. NOS |
| 25K2.00 | 51626 | O/E - abdominal mass - hard |
| 25K3.00 | 97889 | O/E - abdominal mass-very hard |
| R093.00 | 5838 | [D]Swelling, mass or lump within abdomen or pelvis |
| 25J3.00 | 21684 | O/E -abd.mass fills 1 quadrant |
| 14CF.00 | 30630 | History of irritable bowel syndrome |
| 8Cm..00 | 104591 | Management of irritable bowel syndrome |
| J521.11 | 451 | Irritable bowel syndrome |
| J521.00 | 923 | Irritable colon - Irritable bowel syndrome |
| Eu45324 | 16560 | [X]Psychogenic IBS |
| G842z00 | 55438 | Internal haemorrhoids with other complications NOS |
| 7734011 | 49152 | Whitehead haemorrhoidectomy |
| L416300 | 103144 | Haemorrhoids in pregnancy and puerperium with a/n comp |
| G84z.00 | 2096 | Haemorrhoids NOS |
| G845300 | 60688 | External ulcerated haemorrhoids |
| 7736011 | 10848 | Evacuation of thrombosed haemorrhoid |
| Gyu8300 | 98413 | [X]Internal haemorrhoids with other complications |
| 7734000 | 7713 | Haemorrhoidectomy |
| G842200 | 5593 | Internal strangulated haemorrhoids |
| G848100 | 17806 | Prolapsed haemorrhoids NOS |
| G848z00 | 41101 | Haemorrhoids with other complications NOS |
| G842100 | 3178 | Internal prolapsed haemorrhoids |
| G842500 | 102411 | Second degree internal haemorrhoids |
| 7735100 | 39639 | Infrared photocoagulation of haemorrhoid |
| 7734200 | 54354 | Stapled haemorrhoidectomy |
| 7734z00 | 35672 | Excision of haemorrhoid NOS |
| G842000 | 9761 | Internal bleeding haemorrhoids |
| G842400 | 102387 | First degree internal haemorrhoids |
| G845200 | 31506 | External strangulated haemorrhoids |
| G847.00 | 3556 | Thrombosed haemorrhoids NOS |
| G849.00 | 16107 | Residual haemorrhoidal skin tags |
| 7735.00 | 21796 | Destruction of haemorrhoid |
| G842.00 | 30716 | Internal haemorrhoids with other complications |
| 7734100 | 21184 | Partial internal sphincterotomy for haemorrhoid |
| L416100 | 63773 | Haemorrhoids in pregnancy and the puerperium - delivered |
| 7736.00 | 453 | Other operations on haemorrhoid |
| 7735300 | 3199 | Rubber band ligation of haemorrhoid |
| G840.00 | 5763 | Internal haemorrhoids, simple |
| G848300 | 48770 | Ulcerated haemorrhoids NOS |
| G842700 | 103433 | Fourth degree internal haemorrhoids |
| Gyu8400 | 53886 | [X]External haemorrhoids with other complications |
| 7735500 | 96232 | Ligation of haemorrhoidal artery |
| G842300 | 38850 | Internal ulcerated haemorrhoids |
| G845000 | 15257 | External bleeding haemorrhoids |
| 7735200 | 647 | Injection of sclerosing substance into haemorrhoid |
| 7736y11 | 51896 | Mitchell haemorrhoidectomy |
| G84..11 | 3833 | Piles - haemorrhoids |
| 7735z00 | 37535 | Destruction of haemorrhoid NOS |
| G848.00 | 37530 | Haemorrhoids with other complications NOS |
| 7736200 | 38679 | Manual reduction of prolapsed haemorrhoid |
| Gyu8500 | 106050 | [X]Unspecified haemorrhoids with other complications |
| 7735y00 | 36793 | Other specified destruction of haemorrhoid |
| G842600 | 102561 | Third degree internal haemorrhoids |
| G843.00 | 1012 | External haemorrhoids, simple |
| L416200 | 68840 | Haemorrhoids in pregnancy and puerperium - deliv + p/n comp |
| L416.00 | 6560 | Haemorrhoids in pregnancy and the puerperium |
| G846.00 | 26884 | Unspecified simple haemorrhoids |
| G84..00 | 195 | Haemorrhoids |
| 7734y00 | 37528 | Other specified excision of haemorrhoid |
| G845100 | 17626 | External prolapsed haemorrhoids |
| 7736100 | 34190 | Forced manual dilation of anus for haemorrhoid |
| G848200 | 31198 | Strangulated haemorrhoids NOS |
| G841.00 | 29961 | Internal thrombosed haemorrhoids |
| G844.00 | 6045 | External thrombosed haemorrhoids |
| 7735400 | 30544 | Stapling of haemorrhoid |
| 7734.12 | 648 | Haemorrhoidectomy |
| G845z00 | 36778 | External haemorrhoids with other complications NOS |
| L416600 | 29786 | Haemorrhoids in pregnancy |
| G848000 | 2832 | Bleeding haemorrhoids NOS |
| 7734.00 | 20334 | Excision of haemorrhoid |
| L416z00 | 90329 | Haemorrhoids in pregnancy and the puerperium NOS |
| 7736z00 | 36122 | Other operation on haemorrhoid NOS |
| 7736y00 | 36973 | Other specified other operation on haemorrhoid |
| G845.00 | 21811 | External haemorrhoids with other complications |
| 8HkW.00 | 99807 | Referral to chronic fatigue syndrome specialist team |
| R007300 | 1371 | [D]Lethargy |
| 1683.11 | 15516 | C/O - 'tired all the time' |
| F286.11 | 7529 | CFS - Chronic fatigue syndrome |
| F286100 | 97284 | Moderate chronic fatigue syndrome |
| 8HlL.00 | 100414 | Referral for chronic fatigue syndrome activity management |
| F286.14 | 9127 | Post-viral fatigue syndrome |
| R007.00 | 44215 | [D]Malaise and fatigue |
| 168..11 | 6242 | Fatigue - symptom |
| R007z00 | 23932 | [D]Malaise and fatigue NOS |
| 1683.00 | 5751 | Tired all the time |
| E205.12 | 7235 | Tired all the time |
| F286200 | 98734 | Severe chronic fatigue syndrome |
| R007500 | 1147 | [D]Tiredness |
| F286.13 | 27877 | PVFS - Postviral fatigue syn |
| F286.00 | 4546 | Chronic fatigue syndrome |
| 168..12 | 5583 | Lethargy - symptom |
| 1682.00 | 1404 | Fatigue |
| 1684.00 | 17736 | Malaise/lethargy |
| F286.12 | 6190 | Postviral fatigue syndrome |
| R007100 | 1688 | [D]Fatigue |
| F286000 | 98512 | Mild chronic fatigue syndrome |
| Eu46011 | 9656 | [X]Fatigue syndrome |
| 168Z.00 | 29292 | Tiredness symptom NOS |
| 8Q1..00 | 97140 | Activity management for chronic fatigue syndrome |
| 168..00 | 5794 | Tiredness symptom |
| J510700 | 2536 | Diverticulosis of the large intestine NOS |
| J512700 | 50313 | Perforated diverticulum of large intestine NOS |
| J512z00 | 69918 | Perforated diverticulum of intestine NOS |
| J511600 | 51511 | Diverticulitis of the large intestine unspecified |
| J510600 | 53092 | Diverticulosis of the large intestine unspecified |
| J511400 | 72590 | Diverticulitis of the small intestine NOS |
| J511500 | 10483 | Diverticulitis of the colon |
| J510.00 | 3593 | Diverticulosis |
| J512500 | 37813 | Perforated diverticulum of colon |
| J511700 | 62979 | Diverticulitis of the large intestine NOS |
| 7718000 | 34164 | Excision of diverticulum of colon |
| J512600 | 48589 | Perforated diverticulum of large intestine unspecified |
| J51..00 | 8322 | Diverticula of intestine |
| J51z.00 | 9511 | Diverticula of the intestine NOS |
| J510500 | 1101 | Diverticulosis of the colon |
| J511.00 | 347 | Diverticulitis |
| J51..11 | 5857 | Diverticular disease |
| 19FZ.00 | 14695 | Diarrhoea symptom NOS |
| 19F..11 | 192 | Diarrhoea |
| 19F2.00 | 4343 | Diarrhoea |
| Ayu0H00 | 52750 | [X]Diarrhoea+gastroenteritis of presumed infectious origin |
| J525.00 | 5036 | Functional diarrhoea |
| A083.00 | 5090 | Diarrhoea of presumed infectious origin |
| A082000 | 2133 | Dysenteric diarrhoea |
| 19F3.00 | 21294 | Spurious (overflow) diarrhoea |
| J4z..11 | 30321 | Presumed noninfectious diarrhoea |
| J4zz.11 | 17017 | Diarrhoea - presumed non-infectious |
| J521000 | 29835 | Irritable bowel syndrome with diarrhoea |
| Eu45317 | 43316 | [X]Psychogenic diarrhoea |
| A083.11 | 14665 | Diarrhoea & vomiting -? infect |
| E264300 | 15371 | Psychogenic diarrhoea |
| A082100 | 36613 | Epidemic diarrhoea |
| 19F..12 | 1695 | Loose stools |
| E264311 | 10158 | Spurious diarrhoea |
| J43z.11 | 6685 | Chronic diarrhoea |
| J433.11 | 17162 | Dietetic diarrhoea |
| R077100 | 14881 | [D] Stools loose |
| 19F..00 | 5134 | Diarrhoea symptoms |
| 19FZ.11 | 2182 | Diarrhoea & vomiting, symptom |
| J4...13 | 6016 | Noninfective diarrhoea |
| A082z00 | 48313 | Infectious diarrhoea NOS |
| 19G..00 | 7644 | Diarrhoea and vomiting |
| A076.11 | 15289 | Viral diarrhoea |
| J520y00 | 24180 | Other specified constipation |
| E264500 | 15939 | Psychogenic constipation |
| J520.00 | 1709 | Constipation - functional |
| J520400 | 99999 | Chronic constipation |
| J520200 | 25797 | Chronic constipation without overflow |
| 19C..00 | 1028 | Constipation |
| J520000 | 23641 | Acute constipation |
| 19C2.00 | 17652 | Constipated |
| J520100 | 6364 | Chronic constipation with overflow |
| 19CZ.00 | 20450 | Constipation NOS |
| J520z00 | 5803 | Constipation NOS |
| J503100 | 10687 | Faecal impaction |
| 19C..11 | 2004 | Constipation symptom |
| 19EA.00 | 910 | Change in bowel habit |
| R078.00 | 16665 | [D]Change in bowel habit |
| 19EA.11 | 19690 | Altered bowel habit |
| B937X00 | 19130 | Refractory anaemia, unspecified |
| D00yz00 | 9537 | Other specified iron deficiency anaemia NOS |
| D00..11 | 882 | Hypochromic - microcytic anaemia |
| D00..12 | 539 | Microcytic - hypochromic anaemia |
| D001.00 | 21127 | Iron deficiency anaemia due to dietary causes |
| 1271.00 | 8364 | FH: Anaemia |
| D00zz00 | 15439 | Iron deficiency anaemia NOS |
| D21z.00 | 739 | Anaemia unspecified |
| D00..00 | 795 | Iron deficiency anaemias |
| D21z.12 | 1702 | Normocytic anaemia due to unspecified cause |
| D00z200 | 40750 | Idiopathic hypochromic anaemia |
| 2C2Z.00 | 16109 | O/E - anaemia NOS |
| Dyu0000 | 4858 | [X]Other iron deficiency anaemias |
| 1454.00 | 24953 | H/O: anaemia NOS |
| 145..11 | 5833 | H/O: anaemia |
| D21..00 | 3265 | Other and unspecified anaemias |
| D000.00 | 27726 | Iron deficiency anaemia due to chronic blood loss |
| D214.00 | 12176 | Chronic anaemia |
| 2C2..11 | 4952 | O/E - anaemic |
| D0z..00 | 8054 | Deficiency anaemias NOS |
| D000.12 | 48338 | Iron deficiency anaemia due to blood loss |
| 2C22.00 | 43074 | O/E - equivocally anaemic |
| D00y100 | 4839 | Microcytic hypochromic anaemia |
| D00z.00 | 18137 | Unspecified iron deficiency anaemia |
| D00y.00 | 33420 | Other specified iron deficiency anaemia |
| D0...00 | 7841 | Deficiency anaemias |
| 2C2..00 | 15358 | O/E - anaemia |
| 2C23.00 | 15913 | O/E - clinically anaemic |
| 2C24.00 | 15914 | O/E - profoundly anaemic |
| 1451.00 | 31214 | H/O: anaemia - iron deficient |
| 25CZ.00 | 14916 | O/E -abd.pain on palpation NOS |
| R090900 | 16806 | [D]Pain in right iliac fossa |
| 197B.00 | 3978 | Upper abdominal pain |
| R090E00 | 2234 | [D]Recurrent acute abdominal pain |
| R090y00 | 31062 | [D]Other specified abdominal pain |
| 197D.00 | 9695 | Right upper quadrant pain |
| 25C2.00 | 12639 | O/E - abd.pain-R.hypochondrium |
| R090K00 | 8362 | [D]Left upper quadrant pain |
| R090N00 | 19283 | [D]Nonspecific abdominal pain |
| R090100 | 2056 | [D]Abdominal colic |
| R090L00 | 9061 | [D]Left lower quadrant pain |
| R090500 | 542 | [D]Epigastric pain |
| R090P00 | 103540 | [D]Functional abdominal pain syndrome |
| J574800 | 2767 | Rectal pain |
| 25CA.00 | 21583 | O/E - abd. pain - L.iliac |
| Ryu1100 | 52402 | [X]Other and unspecified abdominal pain |
| 1962.00 | 7812 | Colicky abdominal pain |
| 1965.11 | 7306 | Biliary colic symptom |
| 197A.11 | 11070 | General abdominal pain-symptom |
| J41..11 | 23950 | Mucous colitis and/or proctitis |
| R090J00 | 7726 | [D]Right upper quadrant pain |
| R073200 | 28285 | [D]Gas pain (abdominal) |
| 1977.00 | 1181 | Right iliac fossa pain |
| 197C.00 | 22608 | Lower abdominal pain |
| R090z00 | 3338 | [D]Abdominal pain NOS |
| 14C4.00 | 6717 | H/O: colitis |
| 1968.00 | 2383 | Abdominal discomfort |
| 1978.00 | 2982 | Left iliac fossa pain |
| Ryu1000 | 50662 | [X]Pain localized to other parts of lower abdomen |
| 25C3.00 | 19223 | O/E - abd. pain - epigastrium |
| 2I18100 | 13626 | Tenderness of epigastrium |
| 1963.00 | 5691 | Non-colicky abdominal pain |
| R090000 | 16402 | [D]Abdominal tenderness |
| 196..11 | 1976 | Abdominal pain type |
| 25C..15 | 5782 | O/E - abdomen tender |
| J574700 | 3049 | Anal pain |
| R090H00 | 8436 | [D]Upper abdominal pain |
| 1972.00 | 290 | Epigastric pain |
| R090G12 | 7248 | [D] Perineal pain |
| 12E2.00 | 20391 | FH: Colitis |
| R090G00 | 9920 | [D]Pelvic and perineal pain |
| R090.00 | 1763 | [D]Abdominal pain |
| R090400 | 716 | [D]Abdominal cramps |
| J4...11 | 1561 | Colitis - noninfective |
| 197..13 | 5960 | Site of abdominal pain |
| 25C..00 | 15180 | O/E - abdo. pain on palpation |
| R090200 | 1239 | [D]Colic NOS |
| 197A.00 | 24661 | Generalised abdominal pain |
| 1971.00 | 4617 | Central abdominal pain |
| 1A5A.00 | 29400 | C/O perineal pain |
| 1969.00 | 177 | Abdominal pain |
